# Supplementary material for: Saliva TwoStep for rapid detection of asymptomatic SARS-CoV-2 carriers
Source: medRxiv. 2021 Feb 16:2020.07.16.20150250. Preprint. [Version 3] doi: 10.1101/2020.07.16.20150250 (PMC7899473; doi:10.1101/2020.07.16.20150250)
Supplement: 1 [file NIHPP2020.07.16.20150250-supplement-1.pdf]

medRxiv preprint doi: https://doi.org/10.1101/2020.07.16.20150250; this version posted February 16, 2021. The copyright holder for this preprint (which was not certified by peer review) is the author/funder, who has granted medRxiv a license to display the preprint in perpetuity.
It is made available under a CC-BY 4.0 International license .

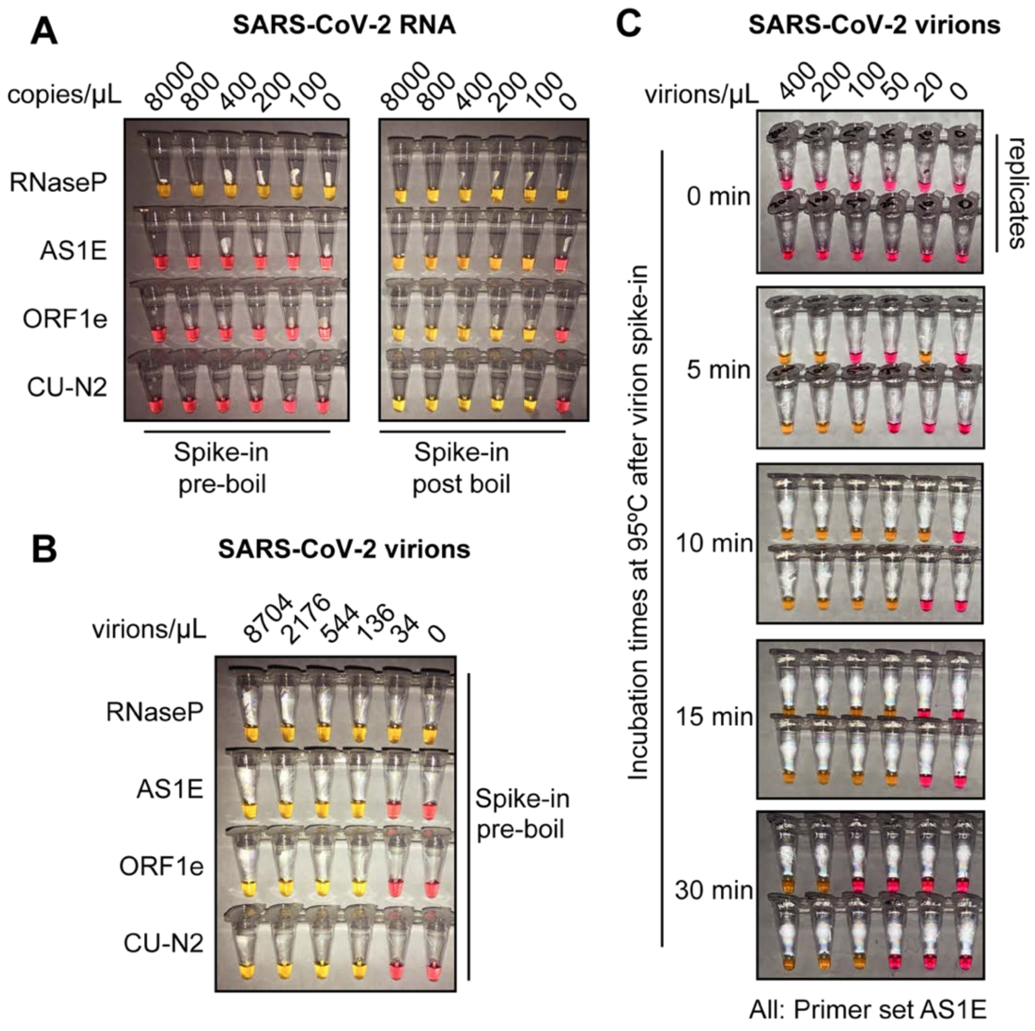

Figure S1: Optimized heat inactivation for safely detecting SARS-CoV-2 in human saliva. A) This experiment shows that heating at 95°C for 10 minutes degrades viral RNA when it is not in the form of virions. Saliva samples were diluted 1:1 with saliva stabilization solution. *In* vitro transcribed SASR-CoV-2 RNA was spiked into the diluted saliva to reach the indicated concentrations before (left) or after (right) the heating at 95°C for 10 minutes. To match other experiments, the indicated concentration represents the copies of SASR-CoV-2 RNA in the original undiluted saliva. The samples were then subjected to RT-LAMP at 65°C for 30 minutes. In this colorimetric version of RT-LAMP, reactions remain pink when no amplification occurred, and turned yellow if there was an amplification event. An RT-LAMP primer set targeting the human RNaseP transcript is included as a host RNA amplification control in addition to the three SARS-CoV-2 primer sets shown in panel A. **B)** This experiment shows that heating saliva at 95°C for 10 minutes does not degrades viral RNA when it is in the form of virions. Saliva samples were spiked with the indicated concentrations of heat-inactivated SARS-CoV-2 virions before being diluted 1:1 with saliva stabilization solution. Samples were then heated at 95°C for 10 minutes and subjected to RT-LAMP similarly to the experiment shown in panel A. **C)** Results illustrate the optimal incubation time at 95°C to liberate SARS-CoV-2 RNA from virions. Saliva samples were spiked with the indicated concentrations of heat-inactivated SARS-CoV-2 virions before being diluted 1:1 with saliva stabilization solution. Samples were then heated at 95°C for the indicated amount of time, and subjected to RT-LAMP similarly to the experiment shown in panel B. Without heating, no SARS-CoV-2 RNA can be detected with RT-LAMP, presumably because virions remain intact and the viral RNA is not accessible by the amplification enzymes. Amplification is somewhat inconsistent at 5 and 30 minutes possibly because at 5 minutes hardly any RNA has been liberated, and by 30 minutes it has been largely degraded. However, 10 or 15 minutes at 95°C appears to provide just the right balance between liberating and preserving RNA. All reactions contain the AS1E primer set. Duplicates are presented at each time point.

medRxiv preprint doi: https://doi.org/10.1101/2020.07.16.20150250; this version posted February 16, 2021. The copyright holder for this preprint (which was not certified by peer review) is the author/funder, who has granted medRxiv a license to display the preprint in perpetuity.
It is made available under a CC-BY 4.0 International license .

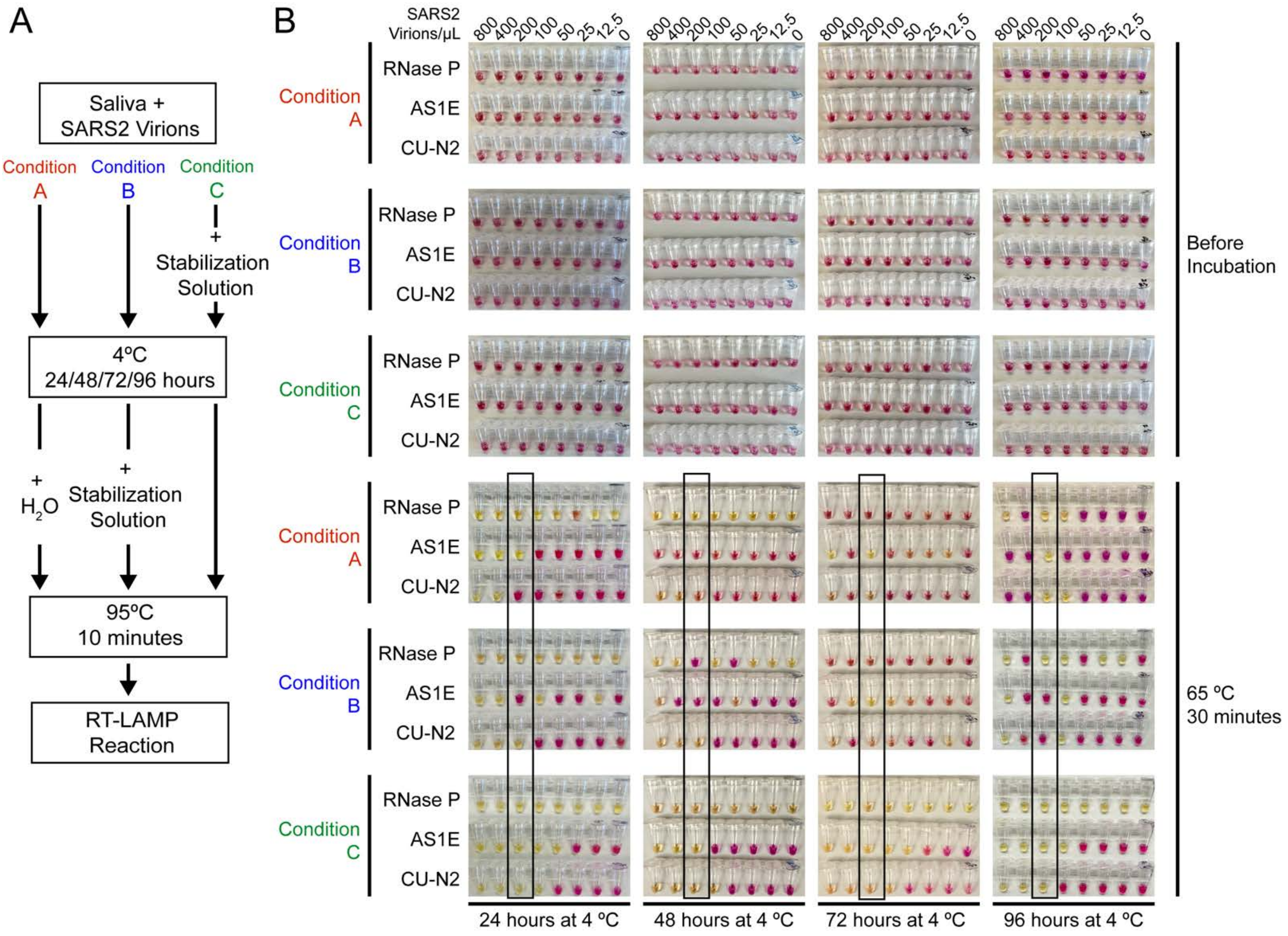

Supplemental Figure S2: Saliva samples are stable at 4ºC for at least 4 days before processing, if stored in saliva stabilization solution. A) Schematic of the experimental conditions. **B)** RT-LAMP reaction result before and after the isothermal amplification. Saliva samples were spiked with heat-inactivated SARS-CoV-2 virions at the indicated concentration and mixed 1:1 with saliva stabilization solution or nuclease-free water before/after storing at 4 ºC for 24, 48, 72 and 96 hours. Samples were then heated at 95ºC for 10 minutes and analyzed using RT-LAMP with the indicated primer sets. Condition C, which is the condition used in our test, performs robustly and is sensitive to the limit of detection even after 96 hours storage at 4ºC. The stated limit of detection of 200 virions/µL is boxed.

medRxiv preprint doi: https://doi.org/10.1101/2020.07.16.20150250; this version posted February 16, 2021. The copyright holder for this preprint (which was not certified by peer review) is the author/funder, who has granted medRxiv a license to display the preprint in perpetuity.
It is made available under a CC-BY 4.0 International license .

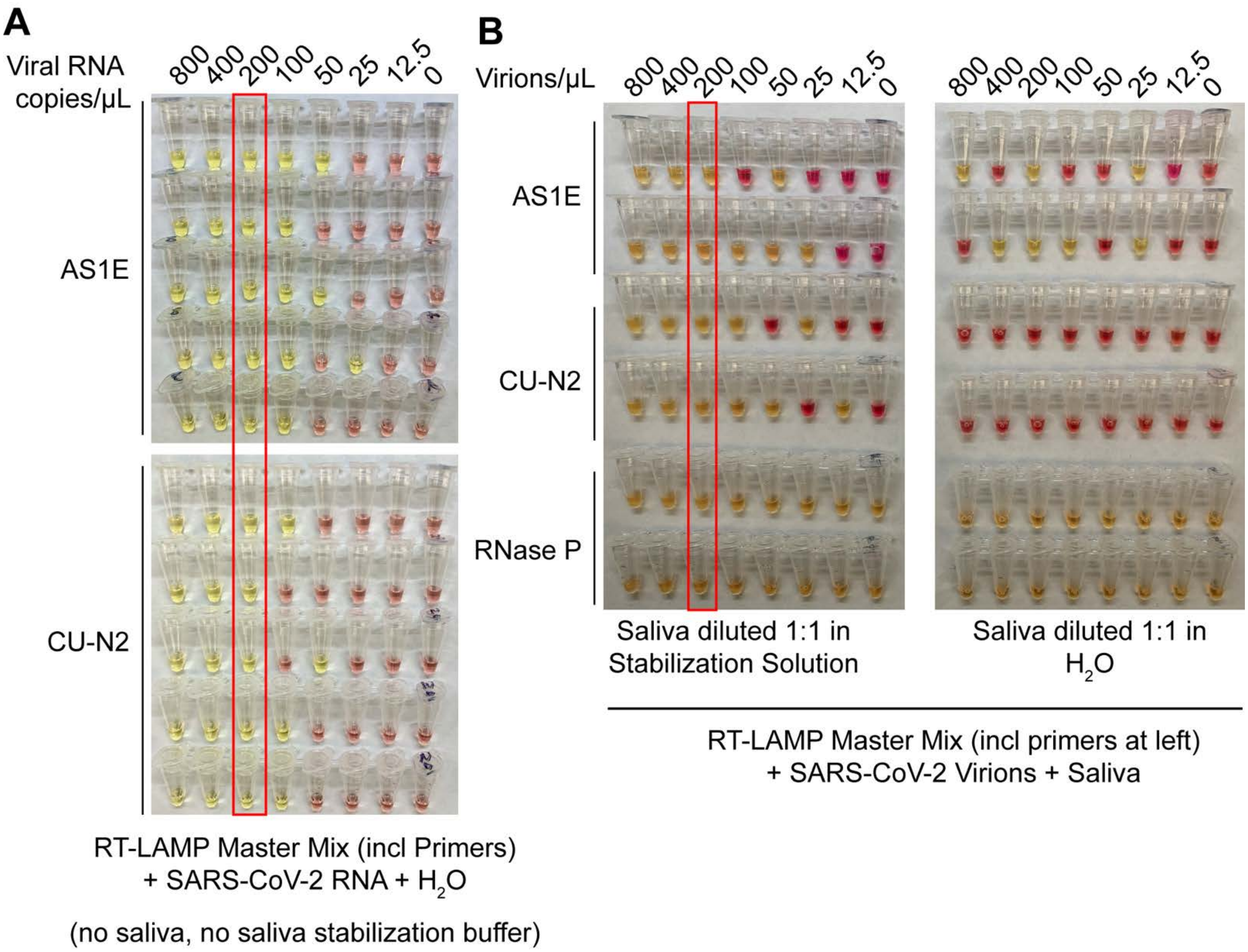

Supplemental Figure S3: Saliva stabilization solution containing NaOH does not lower sensitivity of colorimetric RT-LAMP detection of SARS-COV-2. A) Here, the detection limit of Saliva TwoStep RT-LAMP assay, in the absence of any saliva or saliva stabilization solution, was assessed. This was explored in order to determine whether there might be components of saliva or saliva stabilization solution that inherently lower test sensitivity because they are inhibitory to the RT-LAMP reaction. Here, synthetic SARS-CoV-2 RNA was diluted in nuclease-free water. The diluted RNA was mixed with RT-LAMP reaction mix and incubated at 65ºC for 30 minutes to allow isothermal amplification. Positive reactions turn yellow. Two different primer sets that amplify SARS-CoV-2 were employed, AS1E and CU-N2. The red box indicates the concentration at which positives were identified at least 95% of the time (here, 100% is achieved). That is defined at the limit of detection. Here, it is 200 copies/µL, just as when saliva and saliva stabilization solution is used (see panel B, and data figures in main paper). **B)** Evaluation of RT-LAMP detection limit in the presence of saliva, but in the presence or absence of saliva stabilization solution. Saliva spiked with heat inactivated SARS-CoV-2 virions at specified concentrations was mixed 1:1 with stabilization solution (left) or nuclease-free water (right) and heated at 95ºC for 10 minutes (RNA liberation) before being incubated at 65ºC for 30 minutes (isothermal amplification). On the left, the saliva stabilization solution achieves a limit of detection of 200 virions /µL. When virions are boiled without the saliva stabilization solution (right), very few reactions turn positive and the pattern is unpredictable, presumably because virions and viral RNA are destroyed.

medRxiv preprint doi: https://doi.org/10.1101/2020.07.16.20150250; this version posted February 16, 2021. The copyright holder for this preprint (which was not certified by peer review) is the author/funder, who has granted medRxiv a license to display the preprint in perpetuity.
It is made available under a CC-BY 4.0 International license .

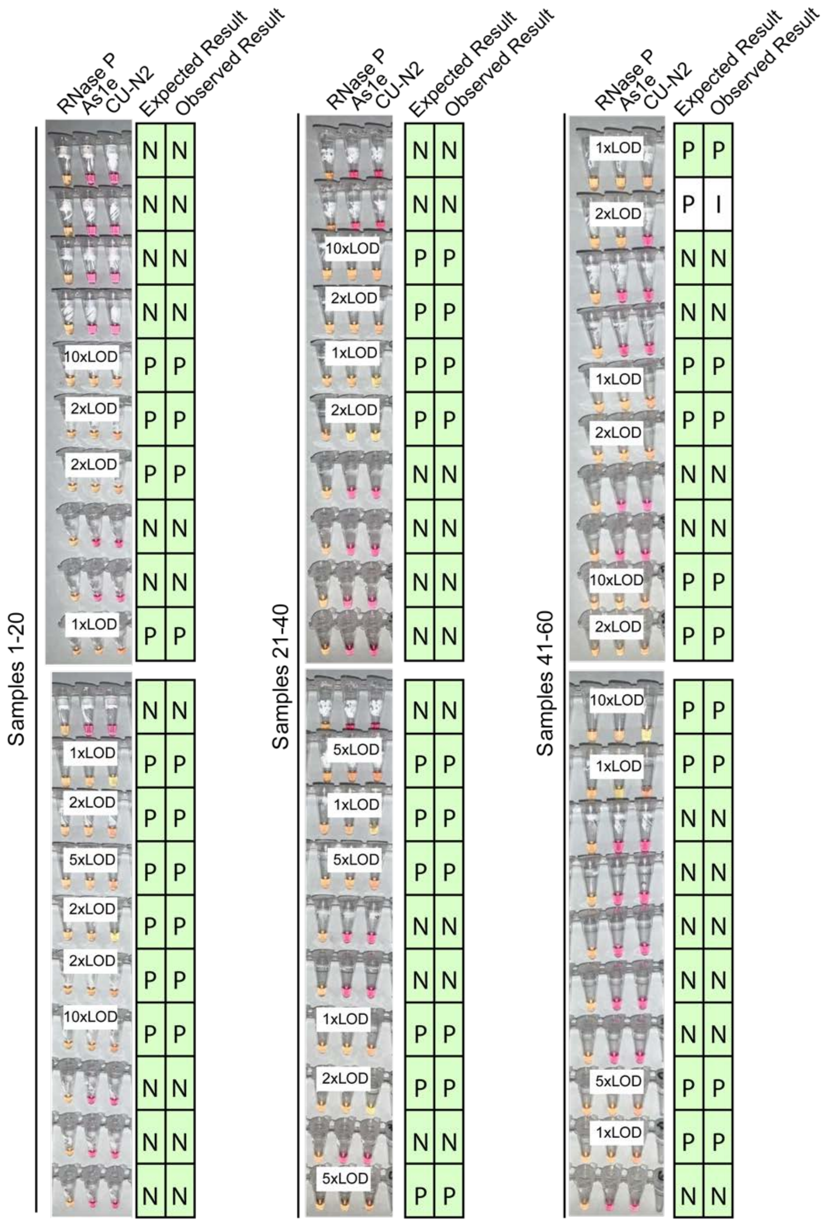

Supplemental Figure S4: Blinded sample evaluation. Plain saliva, or saliva spiked with heat-inactivated SARS-CoV-2 virions at different concentrations, was heated at 95ºC for 10 minutes. Samples were then analyzed using RT-LAMP by a researcher that did not know the true state of each sample. **Experiments in figure)** For each sample, three reactions were performed as indicated by each triplet of tubes. By looking at the patterns of yellow and pink results in each triplet, samples were scored according to the table below. The true status and observed result of each sample are listed to the right (P = Positive, N = Negative, I = Inconclusive). A white box on the triplet is shown if the sample contained SARS-CoV-2. One sample resulted in inconclusive test result. This sample did have SARS-CoV-2 spiked into it, but one of the SARS-CoV-2 primer sets failed to produce a signal (CU-N2). This failed reaction is still pink (negative) even though the tube has 2xLOD virus. 1X LOD = 200 virions/μL. Summary statistics for this experiment are provided in second and third tables below.

Result interpretation

AS1E	CU-N2	RNaseP	Result Interpretation	Report
+	+	+/-	SARS-CoV-2 detected	Positive SARS-CoV-2
If only one of the two targets is positive		+/-	Inconclusive result	Inconclusive
-	-	+	SARS-CoV-2 not detected	Negative SARS-CoV-2
-	-	-	Invalid Result	Invalid

medRxiv preprint doi: https://doi.org/10.1101/2020.07.16.20150250; this version posted February 16, 2021. The copyright holder for this preprint (which was not certified by peer review) is the author/funder, who has granted medRxiv a license to display the preprint in perpetuity.
It is made available under a CC-BY 4.0 International license .

Supplemental Figure S4 (continued)

Summary of results from the contrived specimen study with saliva samples

	virions / µL saliva	Number Tested	RNaseP	AS1E	CU-N2	
1X LOD*	200	9	9 (100%)	9 (100%)	9 (100%)	Positive (%)
2X LOD	400	11	11 (100%)	11 (100%)	10 (91%)	
5X LOD	1000	5	5 (100%)	5 (100%)	5 (100%)	
10X LOD	2000	5	5 (100%)	5 (100%)	5 (100%)	
Negative	0	30	30 (100%)	0 (0%)	0 (0%)	
Total		60				

* 1X LOD (limit of detection = 200 virions/µL)

Summary of positive and negative agreement with contrived saliva samples

		**Contrived Specimen Type**		
		**Positive**	**Negative**	**Total**
**Saliva RT-LAMP Test**	**Positive**	29	0	**29**
	**Inconclusive**	1	0	**1**
	**Negative**	0	30	**30**
	**Total**	**30**	**30**	**60**
**Positive Agreement**		97% (29/30)		
**Negative Agreement**		100% (30/30)		

medRxiv preprint doi: https://doi.org/10.1101/2020.07.16.20150250; this version posted February 16, 2021. The copyright holder for this preprint (which was not certified by peer review) is the author/funder, who has granted medRxiv a license to display the preprint in perpetuity.
It is made available under a CC-BY 4.0 International license .

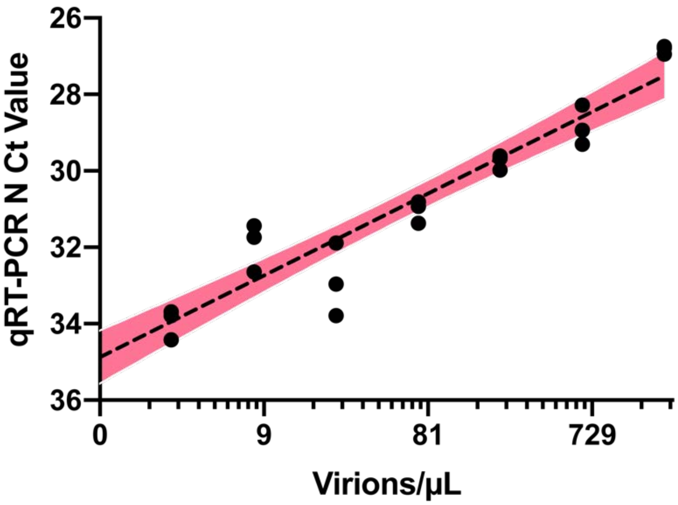

Supplemental Figure S5: Quantitative RT-PCR standard curve used to determine the Ct value to virion/µL calculation. 10,000 copies/µL of heat deactivated SARS-CoV-2 virus was spiked into negative saliva specimens from 6 different volunteers and incubated for 30 minutes at 95ºC. Samples were diluted to indicated concentrations using heat-treated saliva without SARS-CoV-2 addition from the same individual. The standard curve for the primer set targeting SARS-CoV-2 N gene is generated from the linear regression analysis and is illustrated with 95% confidence interval.

medRxiv preprint doi: https://doi.org/10.1101/2020.07.16.20150250; this version posted February 16, 2021. The copyright holder for this preprint (which was not certified by peer review) is the author/funder, who has granted medRxiv a license to display the preprint in perpetuity.
It is made available under a CC-BY 4.0 International license .

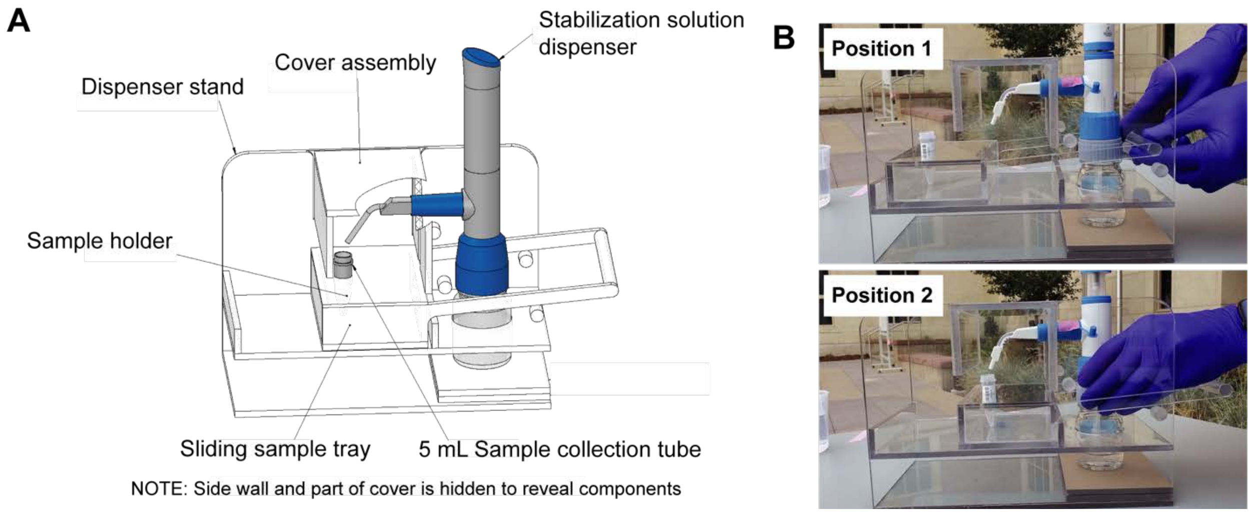

Supplemental Figure S6. Diagrams of the saliva stabilization solution dispensing apparatus. A) CAD model of dispensing apparatus showing components. Custom solution dispensing apparatus fabricated from machined and solvent welded .236in polycarbonate (Tuffak). Polycarbonate is chosen for visibility, strength, and ability to withstand cleaning solvents such as ethanol. This device prevents the need for staff to directly handle uncapped and potentially infectious sample prior to inactivation, limits splash and aerosol exposure risk, and prevents cross-contamination of samples during solution addition step. **B)** Diagram illustrates the operation of the dispensing apparatus. **Position 1:** Tray is extended towards the testing participant and sample tube is seated in tray. Bottle containing stabilization solution and assembled with bottle top dispenser is seated in back section of apparatus. Staff moves tray towards themselves by gently pulling on handle until the tray is seated against back wall of the apparatus. **Position 2:** Sample tray is positioned against the back wall of the apparatus. This brings the sample tube and dispenser nozzle into a set orientation underneath the removable cover assembly. Sample collector uses bottle top dispenser to add 1mL of stabilization solution to sample then pushes sample tray back to the testing participant.

medRxiv preprint doi: https://doi.org/10.1101/2020.07.16.20150250; this version posted February 16, 2021. The copyright holder for this preprint (which was not certified by peer review) is the author/funder, who has granted medRxiv a license to display the preprint in perpetuity.
It is made available under a CC-BY 4.0 International license .

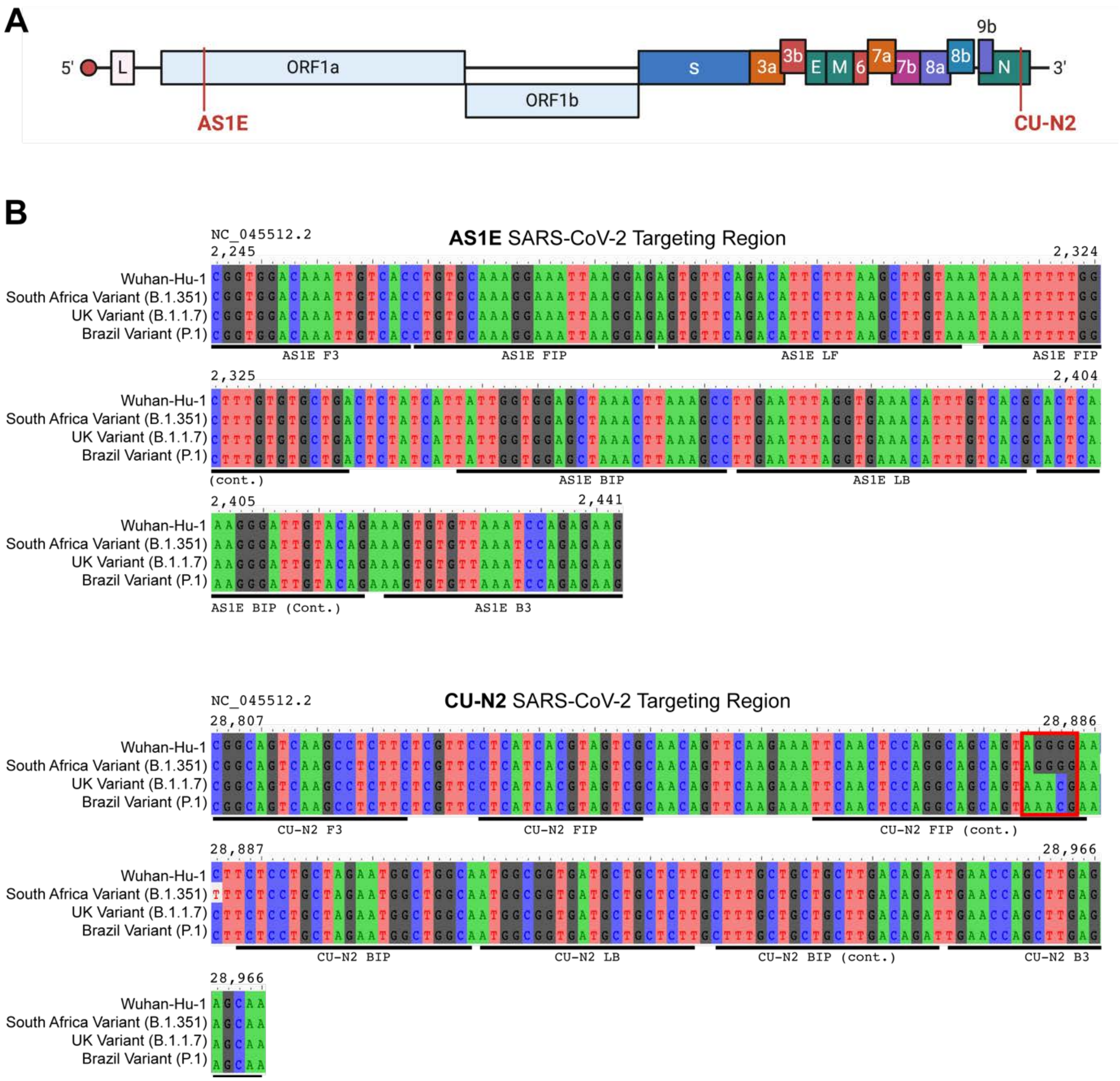

Supplemental Figure S7. Saliva TwoStep primers will detect most or all currently circulating viral variants of concern. A) Genome map of SARS-CoV-2 with the regions targeted RT-LAMP primers highlighted in red. SARS-CoV-2 genome map is adapted from BioRender. **B)** Sequence alignments of regions of the key SARS-CoV-2 genome variants targeted by RT-LAMP primer sets AS1E and CU-N2. Binding regions of each individual primer set component is highlighted in underlying horizontal bars. The SARS-CoV-2 genome region targeted by AS1E primer set is conserved among all variants. For CU-N2, the red box highlights region of sequence variation that might render CU-N2 primer set less effective to identify the UK and Brazil variants. The coordinate of the genome sequence is based on the SARS-CoV-2 reference genome (NCBI NC_045512.2). The SARS-CoV-2 variant representative genomes are downloaded from GISAID (South Africa Variant B.1.351: hCoV-19/South Africa/KRISP-EC-K004572/2020; UK Variant B.1.1.7: hCoV-19/England/MILK-9E2FE0/2020; Brazil Variant P.1: hCoV-19/USA/VA-DCLS-2185/2020).

medRxiv preprint doi: https://doi.org/10.1101/2020.07.16.20150250; this version posted February 16, 2021. The copyright holder for this preprint (which was not certified by peer review) is the author/funder, who has granted medRxiv a license to display the preprint in perpetuity.
It is made available under a CC-BY 4.0 International license .

Supplemental Text S1: Troubleshooting RT-LAMP False-Positives

See also Thi et al (Thi et al., 2020) for additional, excellent troubleshooting advice

Controlling acidic and variable human saliva samples: We found that the biggest obstacle to implementing the colorimetric RT-LAMP assay is the variability in the reaction pH condition. First, false-positive signal can result from saliva samples that are naturally acidic. We spent significant time addressing this issue and ultimately found that all samples must be rendered basic as described in the article in order to set the correct threshold for specificity in the test. This was achieved through a titration series of sodium hydroxide in the saliva stabilization solution to find the optimal concentration that would ensure all RT-LAMP reactions start pink and are still capable of turning yellow if amplification occurs. However, note that other components in the saliva stabilization solution (EDTA, pH=8.0, and TCEP-HCl) have also played a role in establishing the optimal pH. Additional saliva stabilization solution optimization might be needed if other forms of these components are used. Second, the colorimetric RT-LAMP reaction relies on phenol red to detect the pH change during the amplification. Any additional buffering agents, such as tris-acetate or tris-borate that are commonly present in laboratory reagents, should be avoided to prevent potential false negative signals, as these buffering agents tend to inhibit the pH change.

Controlling reaction acidification by carbonic acid: A second issue that has to be carefully controlled is the exposure of reaction components to the surrounding environment. When carbon dioxide from the atmosphere dissolves in water, it creates carbonic acid, which if present in high enough quantities can trigger the phenol red to turn yellow regardless of reaction state. Control measures should be implemented in three ways: First, we advise preparing the reaction mix (RT-LAMP master mix, primers, and water) right before sample loading. This is to prevent background amplification as well as the acidification of the reaction mix due to exposure to air. For this reason, we advise against the use of 96-well plates. Additionally, dry ice should be avoided or completely isolated from the reaction components during sample transportation, as the exposure to the excessive carbon dioxide could also lead to acidification of the reaction mix. Second, during the 30-minute 65ºC incubation, it is essential to completely seal off the reaction tubes to prevent vaporization of the reaction mix, as well as the infiltration of the water vapor if a water bath is used. We have noticed that an incomplete seal could lead to false positive signals. Last, because RT-LAMP amplification is highly robust, the test is very sensitive to contamination (Davidi et al., 2020). Therefore, opening of reaction tubes after RT-LAMP has occurred should be strictly avoided as these tubes contain a large amount of target DNA. Alternatively, the NEB WarmStart LAMP 2X Master Mix with UDG (NEB M1804) can be used to eliminate DNA contamination. Through these results are not shown, we have verified the same limit of detection can be reach using this alternative master mix.

Controlling laboratory-based contamination: (Please also see Davidi et al, 2020 (Davidi et al., 2020)) When carrying out the RT-LAMP SARS-CoV-2 screening test at scale, it is critical to assign isolated workstations, each containing their own set of laboratory equipment such as pipettes, centrifuges, vortexes, and cleaning supplies. This equipment should never move between stations and be regularly decontaminated using a detergent based cleaning solution such as 10% bleach or any other commercially available solution designed to eliminated nucleic acid contaminants. Additionally, special care should be taken by laboratory staff to regularly replace gloves if moving *backwards* from the following workstations:

> **Workstation 1: Setting up master mixes.** This workstation is dedicated to making and aliquoting the master mix containing the RT-LAMP enzymes, primers, and water. All reagents should be centrifuged and spun down after thawing. As mentioned above, master mix should be made and aliquoted shortly before addition of saliva (Workstation 2) to avoid carbon dioxide solubilization due to atmospheric exposure.
>
> **Workstation 2: Adding saliva samples to aliquoted RT-LAMP reaction tubes.** This workstation is dedicated to handling the processed saliva samples (see **Supplemental Text S2** for advice on collecting and processing saliva). Once saliva samples are added to the aliquoted RT-LAMP reaction tubes, care should be taken to ensure that an appropriate seal is established (e.g. dome

medRxiv preprint doi: https://doi.org/10.1101/2020.07.16.20150250; this version posted February 16, 2021. The copyright holder for this preprint (which was not certified by peer review) is the author/funder, who has granted medRxiv a license to display the preprint in perpetuity.
It is made available under a CC-BY 4.0 International license .

cap strips) to minimize airflow during reaction incubation. This workstation should include two micropipettes capable of pipetting a volume of 2 µl. One pipette can be used for saliva samples, while the other pipette should be used exclusively for pipetting any RNA controls (e.g. *in vitro* transcribed SARS-CoV-2 RNA).

Workstation 3: RT-LAMP reaction incubation and results reporting. This workstation contains the heating element (e.g. heat block, thermal cycler) where RT-LAMP reactions are incubated. This Workstation has the highest risk of contamination, since the RT-LAMP reaction products will be in high abundance and can themselves serve as a template in subsequent reactions. When reactions are removed from the heating element, they should immediately be analyzed, results recorded, and then reaction tubes should be disposed in a container with a lid. *Never carry completed reaction tubes to any other part of the lab. Never open completed reaction tubes for any reason.* Any laboratory technician that has entered Workstation 3 should dispose of their gloves before returning to any other part of the lab.

medRxiv preprint doi: https://doi.org/10.1101/2020.07.16.20150250; this version posted February 16, 2021. The copyright holder for this preprint (which was not certified by peer review) is the author/funder, who has granted medRxiv a license to display the preprint in perpetuity.
It is made available under a CC-BY 4.0 International license .

Supplemental Text S2: Recommended procedures for sample collection

When deploying Saliva TwoStep RT-LAMP screening test, it is important to set up saliva sample collection sites that allow large numbers of participants to move through the sample collection process quickly and smoothly. In addition, it is important to exercise extra precautions to avoid sample cross contamination as well as the exposure of the sample or stabilization solution. With this in mind, we have designed and optimized a saliva sample collection workflow that utilizes a customized stabilization dispensing apparatus (**Supplemental Figure S6**):

1. At the designated sample collection site, the screening test participants retrieve a 5 mL screw cap tube (MTC Bio #C2530) and collect passive drool into the 5mL tube until liquid saliva reaches the 1mL graduation mark. Bubbles do not contribute towards the 1mL volume.

2. After collecting saliva, the testing participants submerge capped 5 mL collection tube in a 250mL beaker with 70% ethanol to decontaminate the surface. They then remove the tube, carefully uncap it, and place it in the slot of the dispensing apparatus sample tray while in **Position 1** (**Supplemental Figure S6B**).

3. Staff then move the sample tray towards themselves by gently pulling on sample tray handle until it is seated against the back wall and the sample tube is underneath the cover assembly and centered under the dispenser nozzle (**Supplemental Figure S6B Position 2**). This partially enclosed space limits the potential risk of splashes and aerosols during this solution addition step.

4. Staff then use the bottle top dispenser (Fisher Scientific #13681527) to gently add 1mL of stabilization solution into sample tube (for an approximate 1:1 ratio of sample to solution). The apparatus holds the dispenser nozzle and sample tube in a fixed orientation to prevent cross contamination during this step.

5. Staff slide the sample tray containing the collection tube back towards the participant. The participant re-approaches to cap their sample with the screw-top lid, shakes it vigorously for 5-10 seconds to mix, cleans the surface with a wipe or by dunking in disinfectant, and places it on ice.

6. Staff then heat-inactivates the sample on-site by incubating it in a 95°C water bath or heat block (heat block preferred for minimizing spill risk) for 10 minutes.

7. Before the next testing participant approaches, staff sprays the sample tray with disinfectant.

8. Staff subsequently stores the inactivated sample on ice in a cooler and then transports it to Saliva TwoStep RT-LAMP testing area (see **Supplemental Text S1**).

Biosafety note

Staff involved in saliva collection should wear all appropriate PPE including a fit-tested N95 mask. Regular surface decontamination is performed with 70% ethanol or bleach in the case of spills. Heating elements and cords are secured and situated away from foot traffic. Collection takes place outdoors for ventilation purposes whenever possible. Subjects maintain 10-foot distance from each other while unmasked and producing samples. Hand sanitizer is provided before and after collection procedure. Protocols were approved by the Institutional Biosafety Committee.

Collection site material list:

Staff set up a table with the following supplies: (1) 250mL plastic beaker with 70% ethanol, (1) 250mL plastic beaker with 10% bleach, (2) spray bottles with 70% ethanol, (1) dispenser apparatus (custom polycarbonate device) with bottle top reagent dispenser (Fisher) and 100mL glass bottle with stabilization solution, (1) ice bucket with ice, (1) water bath with tube rack and temperature probe, (1) digital timer, (1)

medRxiv preprint doi: https://doi.org/10.1101/2020.07.16.20150250; this version posted February 16, 2021. The copyright holder for this preprint (which was not certified by peer review) is the author/funder, who has granted medRxiv a license to display the preprint in perpetuity.
It is made available under a CC-BY 4.0 International license .

cooler with ice. Additional items include paper towels, trash receptacles, spill kits, power cords, hand sanitizer, and additional PPE, ethanol, and bleach. Quantities can be scaled up as needed.
